# Supplementary material for: The Genetic Analyses of French Canadians of Quebec Facilitate the Characterization of New Cancer Predisposing Genes Implicated in Hereditary Breast and/or Ovarian Cancer Syndrome Families
Source: Cancers (Basel). 2021 Jul 7;13(14):3406. doi: 10.3390/cancers13143406 (PMC8305212; doi:10.3390/cancers13143406)
Supplement: Supplementary file 1 [file cancers-13-03406-s001.zip › cancers-1247507-supplementary/cancers-1247507-Supplementary-proofreading.pdf]

cancers

MDPI

Supplementary Method: The Genetic Analyses of French Canadians of Quebec Facilitate the Characterization of New Cancer Predisposing Genes Implicated in Hereditary Breast and/or Ovarian Cancer Syndrome families

Caitlin T. Fierheller, Wejdan M. Alenezi and Patricia N. Tonin

1. Literature Review and Wed-Based Searches

PubMed (www.pubmed.ncbi.nlm.nih.gov) was searched for articles between 1 February 2021–28 February 2021 using the following terms: "french"(All Fields) AND ("canadian"[All Fields] OR "canadians"(All Fields)) AND "gene name"(All Fields). Genes investigated include those implicated in hereditary breast cancer (HBC) and/or hereditary breast and ovarian cancer syndromes (HBOC) syndromes: *ATM*, *BARD1*, *BRCA1*, *BRCA2*, *BRIP1*, *CHEK2*, *MLH1*, *MRE11A*, *MSH2*, *MSH6*, *NBN*, *PALB2*, *PMS2*, *PTEN*, *RAD51C*, *RAD51D*, *RECQL*, *STK11*, and *TP53*. Variants that appear in the supplementary tables were identified in BC and/or OC cases in the context of HBC/HBOC from this literature review. Variants were excluded if they were common (minor allele frequency >1%) in non-cancer general population in the Genome Aggregation Database (gnomAD v2.1.1; www.gnomad.broadinstitute.org) [1]), synonymous, or intronic (± 21 nucleotides from the exon).

2. Bioinformatic Tools Used for Evaluating Variants

Variant effect predictor (VEP; grch37.ensembl.org/Homo_sapiens/Tools/VEP?db=core) was used to annotate variants with the following in silico tools:

2.1. Protein damaging

1. Combined Annotation Dependent Depletion (CADD) v1.4 [2]
   - Phred score ≥15 was considered damaging
2. Eigen v1.1 [3]
   - Rankscore ≥0.4 was considered damaging
3. Consensus Deleteriousness (Condel) [4]
   - Rankscore ≥0.4 was considered damaging
4. Meta Logistic Regression (MetaLR) [5]
   - Rankscore ≥0.4 was considered damaging
5. Meta Support Vector Machine (MetaSVM) [5]
   - Rankscore ≥0.4 was considered damaging
6. Protein Variant Effect Analyzer (PROVEAN) v1.1 [6]
   - Rankscore ≥0.4 was considered damaging
7. Rare Exome Variant Ensemble Learner (REVEL) [7]
   - Rankscore ≥0.4 was considered damaging
8. Variant Effect Scoring Tool (VEST) v4.0 [8]
   - Rankscore ≥0.4 was considered damaging

2.2. Conservation

1. Phylogenetic Analysis with Space/Time Models Conservation (phastCons) v1.5 [9]
   - Rankscore ≥0.4 was considered conserved
2. Site-specific Phylogenetic analysis (SiPhy) [10]
   - Rankscore ≥0.4 was considered conserved

3. Phylogenetic P-values (PhyloP) 100 way in vertebrates [11]
    - Rankscore ≥0.4 was considered conserved
4. Genomic Evolutionary Rate Profiling (GERP++) [12]
    - Score ≥2 was considered conserved

2.3. Splicing

1. Database of splicing consensus regions (dbscSNV) adaptive boosting (ADA) [13]
    - Rankscore ≥0.4 was considered conserved to affect splicing
2. dbscSNV random forest (RF) [13]
    - Rankscore ≥0.4 was considered conserved to affect splicing
3. MaxEntScan [14]
    - Difference ≥1 was considered conserved to affect splicing
4. SpliceAI [15]
    - Rankscore ≥0.4 was considered conserved to affect splicing

- The above in silico tools were chosen as the algorithms were shown to have high predictive performance characteristics across different data sets [16].
- Varsome (www.varsome.com) [17] was used to extract the American College of Medical Genetics and Genomics (ACMG) classification (pathogenic, likely pathogenic, uncertain significance, likely benign, benign) and was last accessed on 30 April 2021.
- ClinVar [18] was used to determine the clinical interpretation of identified variants and was last accessed on 30 April 2021.

2.4. Variants in BRCA1 and BRCA2

- Variants in *BRCA1* or *BRCA2* were considered pathogenic if they were classified as pathogenic or likely pathogenic by ACMG or ClinVar (www.ncbi.nlm.nih.gov/clinvar/).
- Variants in *BRCA1* or *BRCA2* were considered potentially pathogenic if they were classified as uncertain significance in ACMG or ClinVar, or with conflicting interpretations in ClinVar (of which at least one submission is uncertain significance).
- BRCAExchange (https://brcaexchange.org) v45 [19] was used to annotate if variants in *BRCA1* or *BRCA2* were considered pathogenic, uncertain significance, or benign based on review from an expert panel.
- National Center for Biotechnology Information (NCBI) Protein (www.ncbi.nlm.nih.gov/protein/) was used to determine the protein domains of *BRCA1* (AAC37594.1) and *BRCA2* (AAB07223.1).
- *BRCA1* and *BRCA2* exon sizes were determined using the University of California Santa Cruz (UCSC; www.genome.ucsc.edu) Genome Browser [20].

2.5. Variants in other genes

- All frameshift and nonsense variants were considered to be potentially pathogenic as they likely result in loss of encoded protein function.
- Missense variants were considered potentially pathogenic if variants were predicted to be damaging in at least four of eight in silico tools and conserved in at least two of four in silico conservation tools described above.
- Splice site variants were considered to affect splicing if they were predicted to do so by at least two out of four in silico tools described above.

References

1. Karczewski, K.J.; Francioli, L.C.; Tiao, G.; Cummings, B.B.; Alföldi, J.; Wang, Q.; Collins, R.L.; Laricchia, K.M.; Ganna, A.; Birnbaum, D.P.; et al. Variation across 141,456 human exomes and genomes reveals the spectrum of loss-of-function intolerance across human protein-coding genes. *bioRxiv* **2019**, 531210, doi:10.1038/s41586-020-2308-7.
2. Rentzsch, P.; Witten, D.; Cooper, G.M.; Shendure, J.; Kircher, M. CADD: predicting the deleteriousness of variants throughout the human genome. *Nucleic Acids Res.* **2019**, *47*, D886–D894, doi:10.1093/nar/gky1016.
3. Ionita-Laza, I.; McCallum, K.J.; Xu, B.; Buxbaum, J. A spectral approach integrating functional genomic annotations for coding and noncoding variants. *Nat. Genet.* **2016**, *48*, 214–220, doi:10.1038/ng.3477.

4. González-Pérez, A.; López-Bigas, N. Improving the assessment of the outcome of nonsynonymous SNVs with a consensus deleteriousness score, *Condel. Am. J. Hum. Genet*. **2011**, *88*, 440–449, doi:10.1016/j.ajhg.2011.03.004.
5. Dong, C.; Wei, P.; Jian, X.; Gibbs, R.; Boerwinkle, E.; Wang, K.; Liu, X. Comparison and integration of deleteriousness prediction methods for nonsynonymous SNVs in whole exome sequencing studies. *Hum. Mol. Genet.* **2015**, *24*, 2125–2137, doi:10.1093/hmg/ddu733.
6. Choi, Y.; Sims, G.E.; Murphy, S.; Miller, J.R.; Chan, A.P. Predicting the Functional Effect of Amino Acid Substitutions and Indels. *PLOS ONE* **2012**, *7*, e46688, doi:10.1371/journal.pone.0046688.
7. Ioannidis, N.; Rothstein, J.H.; Pejaver, V.; Middha, S.; McDonnell, S.K.; Baheti, S.; Musolf, A.; Li, Q.; Holzinger, E.; Karyadi, D.; et al. REVEL: An Ensemble Method for Predicting the Pathogenicity of Rare Missense Variants. *Am. J. Hum. Genet.* **2016**, *99*, 877–885, doi:10.1016/j.ajhg.2016.08.016.
8. Douville, C.; Masica, D.L.; Stenson, P.D.; Cooper, D.N.; Gygax, D.M.; Kim, R.; Ryan, M.; Karchin, R. Assessing the Pathogenicity of Insertion and Deletion Variants with the Variant Effect Scoring Tool (VEST-Indel). *Hum. Mutat.* **2016**, *37*, 28–35, doi:10.1002/humu.22911.
9. Siepel, A.; Bejerano, G.; Pedersen, J.S.; Hinrichs, A.; Hou, M.; Rosenbloom, K.; Clawson, H.; Spieth, J.; Hillier, L.W.; Richards, S.; et al. Evolutionarily conserved elements in vertebrate, insect, worm, and yeast genomes. *Genome Res.* **2005**, *15*, 1034–1050, doi:10.1101/gr.3715005.
10. Garber, M.; Guttman, M.; Clamp, M.; Zody, M.C.; Friedman, N.; Xie, X. Identifying novel constrained elements by exploiting biased substitution patterns. *Bioinform.* **2009**, *25*, i54–i62, doi:10.1093/bioinformatics/btp190.
11. Pollard, K.S.; Hubisz, M.J.; Rosenbloom, K.R.; Siepel, A. Detection of nonneutral substitution rates on mammalian phylogenies. *Genome Res.* **2009**, *20*, 110–121, doi:10.1101/gr.097857.109.
12. Davydov, E.V.; Goode, D.; Sirota, M.; Cooper, G.M.; Sidow, A.; Batzoglou, S. Identifying a High Fraction of the Human Genome to be under Selective Constraint Using GERP++. *PLoS Comput. Biol.* **2010**, *6*, e1001025, doi:10.1371/journal.pcbi.1001025.
13. Jian, X.; Boerwinkle, E.; Liu, X. In silico prediction of splice-altering single nucleotide variants in the human genome. *Nucleic Acids Res.* **2014**, *42*, 13534–13544, doi:10.1093/nar/gku1206.
14. Shamsani, J.; Kazakoff, S.H.; Armean, I.M.; McLaren, W.; Parsons, M.T.; A Thompson, B.; A O'Mara, T.; Hunt, S.; Waddell, N.; Spurdle, A.B. A plugin for the Ensembl Variant Effect Predictor that uses MaxEntScan to predict variant spliceogenicity. *Bioinform.* **2019**, *35*, 2315–2317, doi:10.1093/bioinformatics/bty960.
15. Jaganathan, K.; Panagiotopoulou, S.K.; McRae, J.F.; Darbandi, S.F.; Knowles, D.; Li, Y.I.; Kosmicki, J.A.; Arbelaez, J.; Cui, W.; Schwartz, G.B.; et al. Predicting Splicing from Primary Sequence with Deep Learning. *Cell* **2019**, *176*, 535–548.e24, doi:10.1016/j.cell.2018.12.015.
16. Ghosh, R.; Oak, N.; Plon, S.E. Evaluation of in silico algorithms for use with ACMG/AMP clinical variant interpretation guidelines. *Genome Biol.* **2017**, *18*, 1–12, doi:10.1186/s13059-017-1353-5.
17. Kopanos, C.; Tsiolkas, V.; Kouris, A.; E Chapple, C.; Aguilera, M.A.; Meyer, R.; Massouras, A. VarSome: the human genomic variant search engine. *Bioinform.* **2019**, *35*, 1978–1980, doi:10.1093/bioinformatics/bty897.
18. Landrum, M.J.; Lee, J.M.; Benson, M.; Brown, G.R.; Chao, C.; Chitipiralla, S.; Gu, B.; Hart, J.; Hoffman, D.; Jang, W.; et al. ClinVar: improving access to variant interpretations and supporting evidence. *Nucleic Acids Res.* **2018**, *46*, D1062–D1067, doi:10.1093/nar/gkx1153.
19. Cline, M.S.; Liao, R.G.; Parsons, M.T.; Paten, B.; Alquaddoomi, F.; Antoniou, A.; Baxter, S.; Brody, L.; Cook-Deegan, R.; Coffin, A.; et al. BRCA Challenge: BRCA Exchange as a global resource for variants in BRCA1 and BRCA2. *PLoS Genet.* **2018**, *14*, e1007752, doi:10.1371/journal.pgen.1007752.
20. Kent, W.J.; Sugnet, C.W.; Furey, T.S.; Roskin, K.M.; Pringle, T.H.; Zahler, A.M.; Haussler, D. The Human Genome Browser at UCSC. *Genome Res.* **2002**, *12*, 996–1006, doi:10.1101/gr.229102.
